# Concerns about the interpretation of subgroup analysis

**DOI:** 10.1172/JCI155991

**Published:** 2022-01-18

**Authors:** Arthur M. Albuquerque, Carolina B. Santolia, Ashish Verma

**Affiliations:** 1 School of Medicine, Universidade Federal do Rio de Janeiro, Brazil.; 2 School of Medicine, Universidade Estadual do Rio de Janeiro, Brazil.; 3 Renal Division, Boston University School of Medicine, Boston, Massachusetts, USA.

**Keywords:** COVID-19, Medical statistics

## To the Editor:

We read with interest the article by Li et al. on the association between the use of ACE inhibitors (ACE-Is) and angiotensin receptor blockers (ARBs) and in-hospital mortality among patients with COVID-19 (1). The authors concluded that the use of ARBs was associated with a significant reduction in in-hospital mortality among African American patients but not non–African American patients.

However, we believe this conclusion is not per statistical principles and that it potentially misguides readers. As noted by Altman and Bland (2), statistical analysis should be targeted to the clinical question: is the association between ARB use and in-hospital mortality different between African American and non–African American patients? To answer this question, one should directly compare the estimates (interaction test; ref. 2) performed and reported by the authors. Here we argue that the authors did not accurately interpret this analysis. 

The authors showed an odds ratio (OR) of 0.196 (95% confidence interval [CI] 0.074–0.516) in the African American population and an OR of 0.687 (95% CI 0.427–1.106) in the non–African American population. Accordingly, the interaction term was not significant (95% CI 0.185–1.292; *P* = 0.149; ref. 1). As the authors stated that “Statistical significance was defined as a 2-sided *P* value less than 0.05, unless otherwise stated,” the correct interpretation of this result would be that the association of ACE-I/ARB use and in-hospital mortality was not significantly different between these 2 populations (2). In contrast to this interpretation, the authors concluded that the association was only present in the African American population, which is not compatible with their analysis.

The potential association between ACE-I/ARB use and COVID-19 in-hospital mortality is of great interest to the medical community. Further, the ability to provide reliable subgroup analyses is vital in clinical decision-making (3). Interaction analyses are essential to answer the clinically relevant question of whether a specific subgroup of patients can benefit more from an intervention than another group. However, we believe the correct interpretation of these results does not support the author’s conclusion.

## Version 1 12/02/2021

In-Press Preview

## Version 2 01/18/2022

Electronic publication
